# Rumination in Human Beings

**Published:** 1892-04-09

**Authors:** 


### The Practitioner's Mirror and Retrospect.

[*The Editor will be glad to receive offers of co-operation and contributions from members of the profession. All letters should be addressed to* The Editor, The Lodge, Porchester Square, London, W.]

### MEDICINE.

### RUMINATION IN HUMAN BEINGS.

In ordinary vomiting two sets of muscular movements are combined. There is on the one hand dilation of the cardiac orifice, and on the other contraction of the abdominal walls so as to compress the stomach while the diaphragm is kept rigid. If either set is inoperative, little or no vomiting takes place. If dilation fails, the strongest efforts can do little to empty the stomach, while the gastric contractions, unaided by those of the abdominal walls, are insufficient to do more than to expel some gas or fluid. Both sets of movements are regulated by a nervous centre in the medulla, to which afferent impulses come from various parts of the body. There may be gastric, or ovarian, or cerebral irritation. A poison in the blood may act directly on the centre, or some distant injury indirectly, but in each and all of these ways by which the centre may be set in action the resulting process of vomiting is entirely an involuntary one. Unlike coughing, which can be set in motion by the will, though often involuntary, vomiting is thus purely reflex. Yet there is reason to think that this has not always been the case, and that a process akin to rumination was once carried on at will by our ancestors. Darwin held that at some period or other the process had been under the direction of the will, so that we were then able to eject the contents of the stomach whenever we chose. He noticed that this power of vomiting at will was retained by the simian family amongst others, whilst in some of the lower animals various special functions had been developed on the basis of the vomitory activity. Such are the habit of feeding their young in pigeons and the process of true rumination in so many mammals. The activity which appears under these different forms has become purely reflex in the human species, so much so, that we have to resort to various indirect measures, such as putting the fingers in the throat if we wish to induce vomiting. How we came to lose the power of voluntarily emptying the stomach is not clear, especially as reflex vomiting is so very frequent. Like other late formed organisations, the vomiting centre in its present state is very easily deranged. Hence the frequency of the symptom and its severity in hysteria and insanity. In the human infant vomiting is still largely voluntary; he rejects without retching any excess of milk, and sometimes any unpleasant medicine which is given him. In the adult, cases are not unknown where the voluntary power has been retained, or even where the practice of chewing the cud exists. Since it was first noticed by Fabricius in the 16th century, occasional instances of this reversion to an earlier type have been observed. In some the patient has ruminated regularly and involuntarily. In others there is occasional regurgitation of the food at will. The state of the gastric contents varies greatly; hyperacidity, subacidity, and want of acidity have all been found. In some there were dyspeptic symptoms, in others none. Nor is there evidence of deficient closure of the cardiac end of the stomach. In short, neither in the secretions, nor in the muscular movements of the organ, has anything been discovered capable of affording an explanation other than that given by heredity. Dr. Alt, indeed, thinks that many of these cases are due to hyperacidity, which stimulates peristalsis of the stomach walls. He would treat them by alkalies and by careful feeding. Yet there are certainly instances where there is little or no acid present, and where rumination goes on regularly without nausea or discomfort. A patient of Jürgenson's began to ruminate at ten years of age. Her father and grandfather were found to have done so too. Regurgitation without nausea came on as soon as the meals were ended. Sometimes the patient has been able to stop the habit by a mental effort. In some cases the rumination appears to aid a feeble digestion, the large unmasticated portions of the food returning to the mouth to be crushed and then going back to the stomach. It appears from observations in some of these patients that the pylorus does not open during digestion suddenly, but that the most fluid parts of the food are absorbed or passed through the pyloric ring, and the harder parts retained till near the end of the process.

Dr. Boas regards the complaint as a neurosis of the stomach, but whether it can be traced to anything beyond hereditary disposition is uncertain. It is possible that some of the cases of persistent vomiting in hysterical and other patients are due to tendencies of this kind, but unfortunately no reliable remedy has been discovered. Some patients have broken through the habit from the fear of disgusting their neighbours. In others, some dyspeptic condition has been treated with benefit, but no general rule can be given for management of rumination; nor is it quite certain how far it is desirable to stop it, or how far it is a natural or remedial process. However, the condition is a very rare one, not more than one hundred cases in all having been reported.

